# 713 Acute Change in qSOFA Score as a Prognostic Tool for Diagnosing Sepsis in Burn Patients

**DOI:** 10.1093/jbcr/irad045.189

**Published:** 2023-05-15

**Authors:** Emma Avery, Jane Zhu, Sarah Rehou, Shahriar Sharohki

**Affiliations:** University of Toronto, Toronto, Ontario; University of Toronto, Toronto, Ontario; University of Toronto, Toronto, Ontario; University of Toronto, Toronto, Ontario; University of Toronto, Toronto, Ontario; University of Toronto, Toronto, Ontario; University of Toronto, Toronto, Ontario; University of Toronto, Toronto, Ontario; University of Toronto, Toronto, Ontario; University of Toronto, Toronto, Ontario; University of Toronto, Toronto, Ontario; University of Toronto, Toronto, Ontario; University of Toronto, Toronto, Ontario; University of Toronto, Toronto, Ontario; University of Toronto, Toronto, Ontario; University of Toronto, Toronto, Ontario

## Abstract

**Introduction:**

Sepsis is a diagnostic challenge in all critically ill patients, but particularly so in the burn patient population. The objective of this study was to evaluate if the modified sepsis-3 criteria defined as an acute change in SOFA score ≥2 was predictive of clinical infection.

**Methods:**

The hospital database was reviewed between 2016 and 2019 to identify patients who received broad spectrum antibiotics within 2 days of sustaining their injury. Culture specimens taken at the time of antibiotic administration determined presence of a clinical infection.

**Results:**

Between 2016 and 2019, a total of 98 patients were admitted to the burn unit within 2 days of their injury and received prophylactic meropenum and/or piperacillin/tazobactam for suspicion of clinical infection. When stratified based on an acute change in SOFA score within 48 hours prior to receiving antibiotics, 67 (72%) patients received antibiotics with an acute change in SOFA score ≤1, and 26 (27%) patients received antibiotics with an acute change in SOFA score ≥2. Of those patients with the acute change in SOFA score ≥2, 22 patients (85%) had positive cultures associated with the timing of prophylactic antibiotics, as compared to 60 patients (90%) with a change in SOFA score ≤1 (p value 0.05).

**Conclusions:**

Our data suggests that the modified sepsis-3 criteria is not a reliable tool for diagnosing burn sepsis.

**Applicability of Research to Practice:**

Improving our ability to accurately predict sepsis in burn patients can improve patient outcomes and decrease unnecessary administration of broad spectrum antibiotics.

